# Reference value for expiratory time constant calculated from the maximal expiratory flow-volume curve

**DOI:** 10.1186/s12890-019-0976-6

**Published:** 2019-11-11

**Authors:** Takamitsu Ikeda, Yasuhiro Yamauchi, Kanji Uchida, Koji Oba, Takahide Nagase, Yoshitsugu Yamada

**Affiliations:** 1 Department of Anesthesiology, Graduate School of Medicine, The University of Tokyo, Tokyo, Japan; 2 Department of Respiratory Medicine, Graduate School of Medicine, The University of Tokyo, Tokyo, Japan; 3 Department of Biostatistics, School of Public Health, Graduate School of Medicine, The University of Tokyo, Tokyo, Japan

**Keywords:** Respiratory physiology, Expiratory time constant, Maximal expiratory flow-volume curve, Spirometry, Pulmonary function test

## Abstract

**Background:**

The expiratory time constant (RC_EXP_), which is defined as the product of airway resistance and lung compliance, enable us to assess the mechanical properties of the respiratory system in mechanically ventilated patients. Although RC_EXP_ could also be applied to spontaneously breathing patients, little is known about RC_EXP_ calculated from the maximal expiratory flow-volume (MEFV) curve. The aim of our study was to determine the reference value for RC_EXP_, as well as to investigate the association between RC_EXP_ and other respiratory function parameters, including the forced expiratory volume in 1 s (FEV_1_)/ forced vital capacity (FVC) ratio, maximal mid-expiratory flow rate (MMF), maximal expiratory flow at 50 and 25% of FVC (MEF_50_ and MEF_25_, respectively), ratio of MEF_50_ to MEF_25_ (MEF_50_/MEF_25_).

**Methods:**

Spirometric parameters were extracted from the records of patients aged 15 years or older who underwent pulmonary function testing as a routine preoperative examination before non-cardiac surgery at the University of Tokyo Hospital. RC_EXP_ was calculated in each patient from the slope of the descending limb of the MEFV curve using two points corresponding to MEF_50_ and MEF_25_. Airway obstruction was defined as an FEV_1_/FVC and FEV_1_ below the statistically lower limit of normal.

**Results:**

We retrospectively analyzed 777 spirometry records, and 62 patients were deemed to have airway obstruction according to Japanese spirometric reference values. The cut-off value for RC_EXP_ was 0.601 s with an area under the receiver operating characteristic curve of 0.934 (95% confidence interval = 0.898–0.970). RC_EXP_ was strongly associated with FEV_1_/FVC, and was moderately associated with MMF and MEF_50_. However, RC_EXP_ was less associated with MEF_25_ and MEF_50_/MEF_25_.

**Conclusions:**

Our findings suggest that an RC_EXP_ of longer than approximately 0.6 s can be linked to the presence of airway obstruction. Application of the concept of RC_EXP_ to spontaneously breathing subjects was feasible, using our simple calculation method.

## Background

The expiratory time constant (RC_EXP_) is the parameter that refers to the length of time required for a lung unit to fill or empty. In mechanically ventilated patients, expiration is a passive process that depends on the time constant of the respiratory system, and RC_EXP_ is thus useful for assessing the lung condition to optimize the ventilator settings [1]. Given that RC_EXP_ is defined as the product of airway resistance and lung compliance [2], the variable serves as a dynamic measurement that can reflect the mechanical properties of the respiratory system [3, 4].

The concept of RC_EXP_ is not common in spontaneously breathing patients, but it is possible to calculate it from the maximal expiratory flow-volume (MEFV) curve. According to the equal pressure point theory, the descending limb of the MEFV curve where the maximal expiratory flow is less than 75% of the forced vital capacity (FVC) corresponds to the effort-independent portion [5, 6]. In theory, RC_EXP_ is obtained when maximal expiratory flows at low lung volumes are accurately ascertained, as the slope of the effort-independent portion is known to be expressed as the reciprocal of the time constant of the respiratory system [1, 7]. The maximal expiratory flows measured at a specified point of the MEFV curve can be susceptible to individual variability [8]. Unlike flow-based parameters, however, the value of RC_EXP_ would contain qualitatively different information closely related to respiratory mechanics, given its ability to describe the features of the lung unit with its unique elasticity and capacity to resist airflow.

Standard spirometry, with all its limitations, remains an indispensable tool for detecting airway obstruction, and it can be used as a preoperative screening in patients scheduled for surgery under general anesthesia [9, 10]. Among a range of spirometric parameters, forced expiratory volume in 1 s (FEV_1_)/FVC, maximal mid-expiratory flow rate (MMF), maximal expiratory flows at 50 and 25% of FVC (MEF_50_ and MEF_25_, respectively), and the ratio of MEF_50_ to MEF_25_ (MEF_50_/MEF_25_) are, to one degree or another, linked to the pathology of small airways [8, 11, 12]. On the assumption that RC_EXP_ is also associated with small airways obstruction, it could serve as a marker indicative of intra- or post-operative pulmonary conditions. However, there is no standard for RC_EXP_ calculated from the effort-independent portion of the MEFV curve. The relationship between RC_EXP_ and other spirometric parameters has not been investigated either.

Our study was designed to determine the reference value for RC_EXP_ derived from spirometry, as well as to examine the association between RC_EXP_ and the markers of small airway disease, including FEV_1_/FVC, MMF, MEF_50_, MEF_25_, and MEF_50_/MEF_25_. We then performed a retrospective analysis of our database that contains the records of preoperative spirometry testing obtained from patients scheduled for non-cardiac surgery at the University of Tokyo Hospital, Japan.

## Materials and methods

### Study subjects

Patients scheduled for surgery in Japan are supposed to undergo pulmonary function testing as part of routine preoperative examinations to reveal any undiagnosed respiratory dysfunction. Basically, all patients scheduled for surgery at the University of Tokyo Hospital undergo spirometry testing prior to general anesthesia, under the instruction of the attending doctor. Preoperative pulmonary function measures are occasionally screened in some patients undergoing regional anesthesia to assess their suitability to undergo general anesthesia in case of any sudden change in the type of anesthesia performed. Informed consent was obtained from each patient in advance on the use of data for scientific research.

With the approval of the institutional review board of the University of Tokyo (IRB #11108), we created a database containing information on the pulmonary function of patients scheduled for surgery in order to compare the flow-volume curves obtained prior to and during general anesthesia [13]. This database contains the records of preoperative MEFV curves that were available from patients aged 15 years or older, who were scheduled for non-cardiac surgery under general or regional anesthesia during the period between April 5 and May 31, 2016. A portion of the data, including baseline characteristics of the patients and respiratory function parameters derived from spirometry, had previously been reported [13].

### Quality control of spirometry

In accordance with the guidelines issued by the Japanese Respiratory Society [14], spirometry testing was performed by experienced technicians at our institution to ensure measurement accuracy by diminishing the variability of the results. The acceptability criteria include (1) a continuous maximal effort throughout the maneuver without artefacts, (2) a satisfactory start of expiration with an extrapolated volume of less than 5% of FVC or 150 mL, whichever is larger, and (3) an adequate exhalation with a plateau in the volume-time curve of longer than 2 s, exhalation times of longer than 15 s, or exhalation times of longer than 6 s if the subject cannot continue further exhalation. Acceptable repeatability is achieved when the difference between the largest and the next largest FEV_1_ is within 200 mL of each other and the difference between the largest and the next largest FVC is within 200 mL of each other, after a minimum of three acceptable spirograms have been obtained. The best curve that meets all the criteria above is selected from the usable curves. It also requires that the sum of FEV_1_ and FVC be adequately large in the best curve.

### Study design and methods

Our database of preoperative spirometry testing was retrospectively analyzed to clarify the association between RC_EXP_ and other spirometric parameters, including FEV_1_/FVC, MMF, MEF_50_, MEF_25_, and MEF_50_/MEF_25_, and to estimate the reference value for RC_EXP_. The measured values of FEV_1_/FVC, MMF, MEF_50_, MEF_25_, and MEF_50_/MEF_25_ were extracted from our database. Emphasis was also placed on clarifying the relationship between MMF and other respiratory function parameters sensitive to the degree of airway obstruction in small airways.

### Calculation of RC_EXP_

Based on respiratory mechanics, knowledge on the descending limb of the MEFV curve is described using the following equations:

1 $$ \mathrm{R}=\frac{\mathrm{P}}{\dot{\mathrm{V}}} $$

2 $$ \mathrm{C}=\frac{\mathrm{V}}{\mathrm{P}} $$

where R is airway resistance, C is lung compliance, P is pressure, V is gas volume, and $\dot{\mathrm{V}}$ is air flow. By definition, RC_EXP_ is the product of airway resistance and lung compliance, and is expressed via the eqs. (1) and (2) as:

3 $$ \mathrm{RCexp}=\frac{\mathrm{P}}{\dot{\mathrm{V}}}\times \frac{\mathrm{V}}{\mathrm{P}}=\frac{\mathrm{V}}{\dot{\mathrm{V}}} $$

**Fig. 1:**
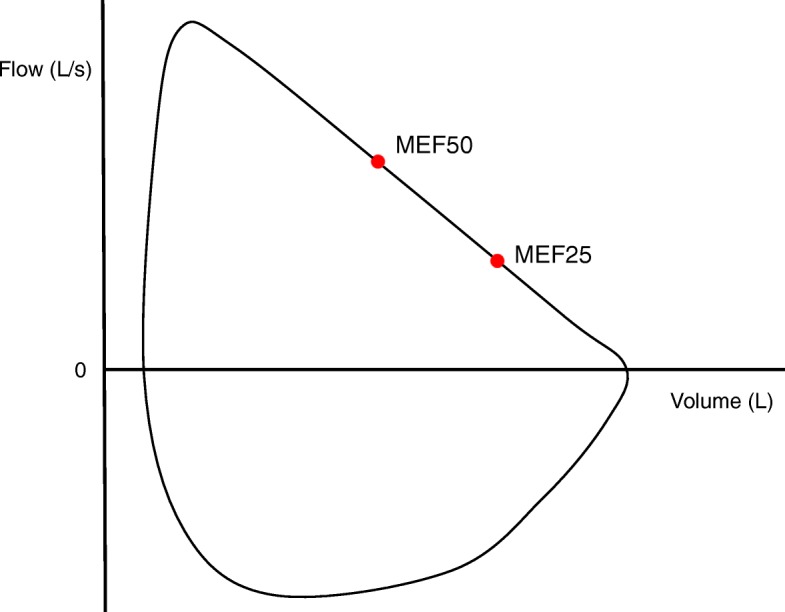
Visual representation of maximal expiratory flow at 50% (MEF_50_) and 25% (MEF_25_) of forced vital capacity (FVC). The MEF_50_ and MEF_25_ are shown as red points located along the descending limb of the maximum expiratory flow-volume (MEFV) curve. The expiratory time constant (RC_EXP_) is calculated as the reciprocal of the slope of the line passing through MEF_50_ and MEF_25_

The eq. (3) refers to RC_EXP_ as the reciprocal of the slope of the descending limb. RC_EXP_ is theoretically obtained when two points along the effort-independent part of the descending limb, MEF_50_ and MEF_25_ for instance, are ascertained. In the present study, the value of RC_EXP_ was calculated as the reciprocal of the slope of the line passing through the two points corresponding to MEF_50_ and MEF_25_ (Fig. 1) by using the following equation:

$$ \mathrm{RCexp}=\frac{0.25\mathrm{FVC}}{\mathrm{MEF}50-\mathrm{MEF}25} $$

### Statistical analysis

Data are expressed as mean ± standard deviation, median and interquartile range, or n (%). The R^2^ value was calculated for the relationships between the spirometric parameters examined. The receiver operating characteristic (ROC) curve was generated for RC_EXP_ to select the cut-off value in accordance with the presence of airway obstruction that was defined as a FEV_1_/FVC ratio and FEV_1_ below the statistically lower limit of normal (LLN) [15, 16]. The area under the ROC curve (AUC) was also obtained for RC_EXP_.

All statistical analyses were performed using EZR (Saitama Medical Center, Jichi Medical University, Saitama, Japan), a graphical user interface for R (The R Foundation for Statistical Computing, Vienna, Austria), which is precisely described as a modified version of R commander designed to add statistical functions used in biostatistics [17]. A *P* value of < 0.001 was considered statistically significant.

## Results

Overall, 777 patients aged 15 years or older were scheduled for surgery and underwent preoperative spirometry testing between April 5 and May 31, 2016. Of the patients scheduled for non-cardiac surgery, 689 underwent general anesthesia and 88 underwent regional anesthesia. The characteristics of the patients are summarized in Table 1. Airway obstruction was defined as an FEV_1_/FVC and FEV_1_ below their respective LLN values. When using Japanese spirometric reference values [18], 62 patients were deemed to have airway obstruction in the present study.

**Table 1:** Baseline characteristics and respiratory function parameters obtained from spirometry

Age (years)	59.28 ± 15.89
Sex (male), n (%)	385 (49.55%)
Height (cm)	160.93 ± 9.04
Body weight (kg)	61.66 ± 28.79
Body mass index	23.68 ± 9.66
FEV_1_ (L)	3.26 ± 13.42
	2.42 (2.00–2.90)
VC (L)	3.25 ± 0.82
	3.17 (2.68–3.79)
FVC (L)	3.21 ± 0.82
	3.15 (2.63–3.74)
FEV_1_/FVC (%)	77.22 ± 9.63
	77.78 (71.40–83.34)
MMF (L/s)	2.27 ± 1.15
	2.10 (1.40–3.00)
MEF_50_ (L/s)	3.01 ± 1.33
	2.88 (2.00–3.90)
MEF_25_ (L/s)	0.92 ± 0.66
	0.74 (0.46–1.23)
MEF_50_/MEF_25_	3.96 ± 1.57
	3.75 (2.88–4.74)
RC_EXP_ (s)	0.48 ± 0.38
	0.40 (0.31–0.53)

Data are expressed as mean ± standard deviation, median and interquartile range, or n (%)

The body mass index is the weight in kilograms divided by the square of the height in meters

*FEV*_1_ forced expiratory volume in 1 s

*VC* vital capacity

*FVC* forced vital capacity

*MMF* maximal mid-expiratory flow rate

*MEF*_50_ maximal expiratory flows at 50% of FVC

*MEF*_25_ maximal expiratory flows at 25% of FVC

*MEF*_50_/*MEF*_25_ the value of MEF_50_ divided by that of MEF_25_

*RC*_*EXP*_ expiratory time constant

**Fig. 2:**
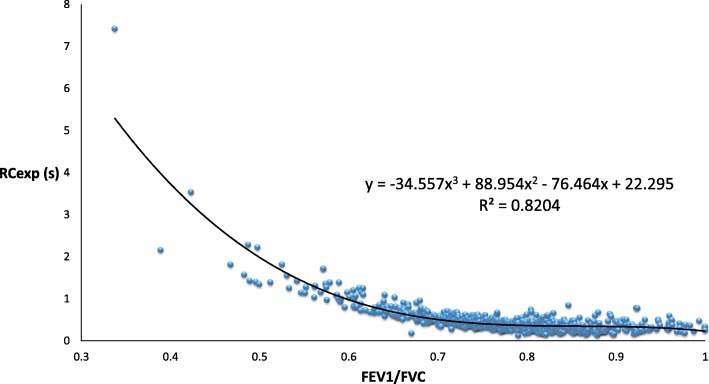
The relationship between expiratory time constant (RC_EXP_) and forced expiratory volume in 1 s/forced vital capacity (FEV_1_/FVC). The value of RC_EXP_, which is calculated based on the effort-independent part of the MEFV curves, is closely associated with FEV_1_/FVC, with a high R^2^ value of 0.8204 (*P* < 0.001). Notably, there is a substantial increase in RC_EXP_ with an FEV_1_/FVC ratio being less than approximately 0.70

**Fig. 3:**
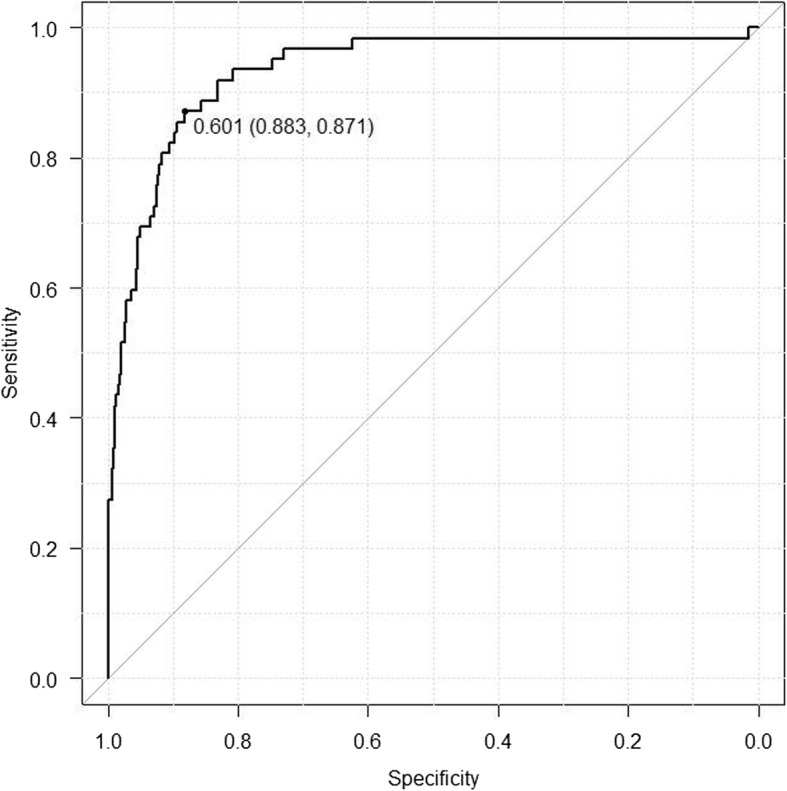
The receiver operating characteristic (ROC) curve for RC_EXP_. With airway obstruction being defined as an FEV_1_/FVC and FEV_1_ below the statistically lower limit of normal, the cut-off value for RC_EXP_ is 0.601 s. The area under the receiver operating characteristic curve (AUC) is calculated as 0.934 (95% confidence interval [CI] = 0.898–0.970)

The relationships between RC_EXP_ and the spirometric parameters, including FEV_1_/FVC, MMF, MEF_50_, MEF_25_, and MEF_50_/MEF_25_, are displayed as scatter plots (Fig. 2, Additional file 1: Figures S1 – S4), each with an R^2^ value of 0.8204, 0.3154, 0.4933, 0.1172, and 0.0144, respectively. The cut-off value for RC_EXP_ was 0.601 s with an AUC of 0.934 (95% confidence interval = 0.898–0.970) (Fig. 3). The relationships between MMF and expiratory flow at lower lung volumes are also displayed as scatter plots (Additional file 1: Figures S5 and S6). MMF was closely associated with both MEF_50_ and MEF_25_ with an R^2^ value of 0.9005 and 0.8885, respectively.

## Discussion

### Main findings

Our retrospective analysis of 777 patients who underwent pulmonary function testing prior to surgery at our institution revealed that the cut-off value for RC_EXP_ calculated from the MEFV curves was 0.601 s with an AUC of greater than 0.9. Among the spirometric parameters that are presumed to predict peripheral airways dysfunction, RC_EXP_ was strongly or moderately associated with FEV_1_/FVC, MMF, and MEF_50_, whereas it was less associated with MEF_25_ and MEF_50_/MEF_25_. Even in spontaneously breathing subjects, calculation of RC_EXP_ was possible using the descending limb of the MEFV curve, and our findings imply that a prolonged RC_EXP_, especially if it is longer than 0.601 s, could be associated with airway obstruction.

### Physiological interpretation of expiratory time constant

To our knowledge, this study is the first attempt to provide the reference value for RC_EXP_ that was calculated from the effort-independent portion of the MEFV curve. In theory, RC_EXP_ can be altered depending on the degree of airway obstruction in spontaneously breathing patients. The finding that most patients without airway obstruction had an RC_EXP_ of shorter than 0.6 s would be comparable to that of a previous study by McIlroy et al., who reported an average time constant of 0.38 s (ranging from 0.28 to 0.51 s) in their healthy, non-intubated subjects [7]. As might be expected, however, our reference value for RC_EXP_ did not exceed the time constant values in mechanically ventilated patients with acute respiratory distress syndrome, which was reported to be in the range of 0.60 to 0.70 s [19, 20].

McIlroy et al. employed the slope of the line drawn using exhaled tidal volume and flow to determine values of the time constant of a relaxed expiration [7]. The reason why their findings were in agreement with those obtained during forced expiration in our study population could be attributable to the mechanism by which forced expiration is governed. As demonstrated in the comparison between relaxed and forced expirations in the same subject, the time rate of change in volume was similar under the relaxed and forced conditions [21]. Even when a greater expiratory flow is achieved, RC_EXP_ will not be shorter than that during relaxed expiration, as the ratio of volume to flow is similar because of the difference in volume expired during forced and relaxed expirations [7]. As long as the linearity of the expiratory flow-volume curves validates the assumption that the linear portion is indicative of the mechanical properties of the respiratory system, namely its resistance and compliance, the value of RC_EXP_ remains theoretically unchanged irrespective of whether the phase of expiration ends at the residual volume or at the functional residual volume.

In contrast, the finding that RC_EXP_ gradually increased in tandem with the decrease in FEV_1_/FVC, MMF, and MEF_50_, especially when they were decreased below a certain level, could be interpreted as collateral evidence for the uneven distribution of RC_EXP_ in patients with airway obstruction [22]. In a model resembling a lung unit where a single elastic element passively empties through a tube open to the atmosphere, the amount of ventilation depends on the compliance of the element and the resistance of the tube. When a particular portion of the lung unit is inadequately ventilated because of the narrowing of its airway, the increase in its airway resistance results in a prolonged RC_EXP_ [23]. This is because the expiratory flow of emptying such a unit is determined using its time constant, the product of its airway resistance and lung compliance. The inequality of ventilation would therefore be a possible mechanism underlying the decreased rate of emptying of lung units with a larger airway resistance, the degree of which could be expressed as a longer RC_EXP_ observed with an increase in the proportion of poorly ventilated regions. On the basis of our previous finding that patients with an FEV_1_/FVC ratio of less than 0.70 showed a substantial increase in the calculated value of airway resistance prior to general anesthesia [13], it could be inferred that elevated airway resistance was a major contributor to the increase in RC_EXP_.

In the present study, we calculated RC_EXP_ by dividing a quarter of the FVC by the gap between MEF_50_ and MEF_25_. Even in healthy subjects, a degree of variability can exist in the parameters available from spirometry, partly because of the variability in FVC values that are possibly influenced by expiratory effort [8]. The advantage of our calculation method would lie in minimizing the variability in FVC, MEF_50_, and MEF_25_, thereby leading to decreased standard deviations of RC_EXP_. Even then, it would still be difficult to simply extrapolate the concept of the linearity of the flow-volume relationship to curvilinear MEFV curves scooping in toward the volume axis, considering that the MEF_50_/MEF_25_ ratio, which could be related to non-homogeneous emptying of the lung, was not constant regardless of the degree of airway obstruction.

### MMF and expiratory flow at lower lung volumes

Given the phenomenon of maximal expiratory flow in which the equal pressure point shifts along the downstream segment to more peripheral airways and is eventually established in non-cartilaginous airways that are easily collapsible [24], the maximal expiratory flows measured at the lower range of FVC are likely sensitive to increased peripheral airway resistance where expiratory flow limitation occurs [25, 26]. For this reason, the measures derived from the middle or latter aspect of the MEFV curve, including MMF, MEF_50_, and MEF_25_, has been regarded as surrogate markers of peripheral airways obstruction.

The finding that there was a highly positive correlation between MMF and MEF_50_ is in close agreement with the finding of Bar-Yishay et al., who analyzed MEFV curves obtained from a large sample of children [27]. MMF is a time-weighted average flow over the mid-vital capacity range, and it is, by definition, likely that MMF contains information that is responsible for the physiological events occurring at the middle aspect of the MEFV curve. On the assumption that the lung empties non-homogeneously with more than a single time constant, the difference between MMF and MEF_50_ would theoretically reflect the degree of airway obstruction [28]. However, Bar-Yishay et al. presented the evidence that the ratio of MEF_50_ to MMF was not affected by peripheral airways obstruction, suggesting the possibility that this ratio is less reflective of the curvilinearity MEFV curve [27]. Their conclusion was that reporting both MMF and MEF_50_ was redundant, considering the close correlation between them. There would nevertheless be value in reporting MEF_25_, as it appeared from our study that the relationship between MMF and MEF_25_ was rather quadratic than simply linear. This might be because of the qualitative difference between MEF_50_ and MEF_25_ in the ability to detect airway obstruction, although both are supposed to surrogate markers of early small airway disease. The finding that RC_EXP_ was less associated with MEF_25_ than with MEF_50_ might also be related to the different property of MEF_25_.

MEFV curve evaluation using the slope-ratio (SR) index, which quantifies the instantaneous slope at any point along the MEFV curve, allows for assessment of special changes in curvature over a range of lung volumes [2]. It also provides additional information that is overlooked by the evaluation of MEFV curves based on absolute and relative values of volume and flow [29]. In elderly healthy subjects, there is a steady increase in SR with the progression of expiration [30], and consequently the decrease in expiratory flow occurs mainly at lower lung volumes [31]. The SR analysis used to detect difference in MEFV curves due to mild chronic obstructive pulmonary disease has demonstrated that the late scooping observed in these subjects is indicative of the normative aging process [29]. The interpretation of decreased MEF_50_ and MEF_25_ should thus be made with caution especially in older subjects.

### MEF_50_/MEF_25_

MEF_50_/MEF_25_ is occasionally used in Japan to evaluate the degree of airway obstruction [12, 32]. Patients with airway obstruction frequently exhibit a marked decrease in MEF_25_ compared with MEF_50_, resulting in an increase in MEF_50_/MEF_25_ [32]. Some studies have suggested that an elevated MEF_50_/MEF_25_ is associated with the pathology of small airways, especially when it is greater than 4.0 [32, 33], but whether MEF_50_/MEF_25_ functions as a marker of small airway disease is still obscure because of the lack of sufficient epidemiological data for this parameter.

A Japanese study reported that MEF_50_/MEF_25_ was greater than 4.0 in many healthy subjects aged 40 years or older, with no difference in MEF_50_/MEF_25_ between smokers and non-smokers, suggesting that it could be difficult to detect the presence of airway obstruction using only MEF_50_/MEF_25_ [8]. This tendency was consistent with our results in which MEF_50_/MEF_25_ exceeded 4.0 in more than one-third of the study population without airflow obstruction. The limited utility of MEF_50_/MEF_25_ may be explained by the qualitative difference between MEF_50_ and MEF_25_ in the degree of association with small airway pathology. MEF_50_/MEF_25_ could nevertheless be useful in younger subjects, as healthy adults aged 30 years or younger generally have a MEF_50_/MEF_25_ of less than 3.0 [32].

### Limitations

Several limitations of our study should be mentioned. First, it was not clarified whether RC_EXP_ was more sensitive than other spirometric parameters in detecting the pathology of small airways. Our results showed that the value of RC_EXP_ quantified from spirometry was associated with airway obstruction, but it was unclear whether RC_EXP_ could provide more useful clinical information than standard spirometric measures. It would be necessary to explore the extent to which RC_EXP_ reflects the different level of severity of airway obstruction because there was a limited number of patients with airway obstruction in our study population. Second, this is a retrospective study and the quality of spirometry testing performed in our patients may be questioned. Improved quality and standardization of forced expiratory maneuver is required to properly interpret the results. Every possible attempt was made to ensure quality-assured and standardized spirometry at our institution. Third, it was difficult to assess the effect of cigarette smoking on lung function because current and former smokers were included in our study. As reported before, an age-related decline has been noted in the maximal expiratory flows in the smoking population aged 40 years or older [8]. Finally, we included only Japanese patients scheduled for surgery under general or regional anesthesia. Although our results cannot be simply applied to different races other than Asians, our reference value for RC_EXP_ could still be theoretically useful in assessing the degree of airway obstruction if it reflects the properties of the respiratory system.

## Conclusions

Our study shed light on the calculated value of RC_EXP_ that was derived from the effort-independent portion of the MEFV curve, suggesting that an RC_EXP_ of longer than approximately 0.6 s can be linked to the presence of airway obstruction in spontaneously breathing patients. While monitoring of RC_EXP_ allows us to assess the overall respiratory mechanics in critical care practice, it would be feasible to apply the concept of RC_EXP_ to non-intubated subjects, using our simple method of calculating RC_EXP_ from the MEFV curve. Further studies are warranted to confirm the ability of RC_EXP_ to detect the presence of airway obstruction.

## Supplementary information

**Additional file 1: Figure S1.** The relationship between RC_EXP_ and maximal mid-expiratory flow rate (MMF). To a certain extent, RC_EXP_ is sociated with MMF with an R^2^ value of 0.3154 (*P* < 0.001). There is a gradual increase in RC_EXP_, especially when MMF is below approximately 1.0 L/s. **Figure S2.** The relationship between RC_EXP_ and maximal expiratory flow at 50% of FVC (MEF_50_). MEF_50_ is one of the spirometric parameters used to calculate RC_EXP_. RC_EXP_ is moderately associated with MEF_50_ with an R^2^ value of 0.4933 (*P* < 0.001). When MEF_50_ is below approximately 1.5 L/s, RC_EXP_ increases with a reduction in MEF_50_. **Figure S3.** The relationship between RC_EXP_ and maximal expiratory flow at 25% of FVC (MEF_25_). MEF_25_ is also one of the spirometric parameters used to calculate RC_EXP_. As compared with MEF_50_, RC_EXP_ is less associated with MEF_25_, and the R^2^ value was estimated to be 0.1172 (*P* < 0.001). **Figure S4.** The relationship between RC_EXP_ and maximal expiratory flow at 50% of FVC divided by maximal expiratory flow at 25% of FVC (MEF_50_/MEF_25_). Overall, RC_EXP_ is almost constant regardless of the value of MEF_50_/MEF_25_. As compared with MEF_50_ and MEF_25_, RC_EXP_ is less associated with MEF_50_/MEF_25_ with an R^2^ value of 0.0144 (*P* = 0.001331). **Figure S5.** The relationship between MMF and MEF_50_. Both MMF and MEF_50_ are parameters that quantify flow in the middle portion of the descending limb of the MEFV curve. MMF is linearly associated with MEF_50_ with a high R^2^ value of 0.9005. **Figure S6.** The relationship between MMF and MEF_25_. MMF is also closely associated with MEF_25_, and there is an almost linear relationship when MMF is below approximately 3.0 L/s.

## Data Availability

The datasets used and analyzed during the current study may be made available from the corresponding author on reasonable request.

## Abbreviations

FEV_1_: forced expiratory volume in 1 s
FVC: forced vital capacity
MEF_25_: maximal expiratory flows at 25% of FVC
MEF_50_: maximal expiratory flows at 50% of FVC
MEF_50_/MEF_25_: ratio of MEF_50_ to MEF_25_
MEFV: maximal expiratory flow-volume
MMF: maximal mid-expiratory flow rate
RC_EXP_: expiratory time constant
VC: vital capacity
