# Supplementary material for: Reference value for expiratory time constant calculated from the maximal expiratory flow-volume curve
Source: BMC Pulm Med. 2019 Nov 11;19:208. doi: 10.1186/s12890-019-0976-6 (PMC6849182; doi:10.1186/s12890-019-0976-6)
Supplement: Supplementary file 1 — Additional file 1: Figure S1. The relationship between RCEXP and maximal mid-expiratory flow rate (MMF). To a certain extent, RCEXP is sociated with MMF with an R2 value of 0.3154 (P < 0.001). There is a gradual increase in RCEXP, especially when MMF is below approximately 1.0 L/s. Figure S2. The relationship between RCEXP and maximal expiratory flow at 50% of FVC (MEF50). MEF50 is one of the spirometric parameters used to calculate RCEXP. RCEXP is moderately associated with MEF50 with an R2 value of 0.4933 (P < 0.001). When MEF50 is below approximately 1.5 L/s, RCEXP increases with a reduction in MEF50. Figure S3. The relationship between RCEXP and maximal expiratory flow at 25% of FVC (MEF25). MEF25 is also one of the spirometric parameters used to calculate RCEXP. As compared with MEF50, RCEXP is less associated with MEF25, and the R2 value was estimated to be 0.1172 (P < 0.001). Figure S4. The relationship between RCEXP and maximal expiratory flow at 50% of FVC divided by maximal expiratory flow at 25% of FVC (MEF50/MEF25). Overall, RCEXP is almost constant regardless of the value of MEF50/MEF25. As compared with MEF50 and MEF25, RCEXP is less associated with MEF50/MEF25 with an R2 value of 0.0144 (P = 0.001331). Figure S5. The relationship between MMF and MEF50. Both MMF and MEF50 are parameters that quantify flow in the middle portion of the descending limb of the MEFV curve. MMF is linearly associated with MEF50 with a high R2 value of 0.9005. Figure S6. The relationship between MMF and MEF25. MMF is also closely associated with MEF25, and there is an almost linear relationship when MMF is below approximately 3.0 L/s. [file 12890_2019_976_MOESM1_ESM.pdf]

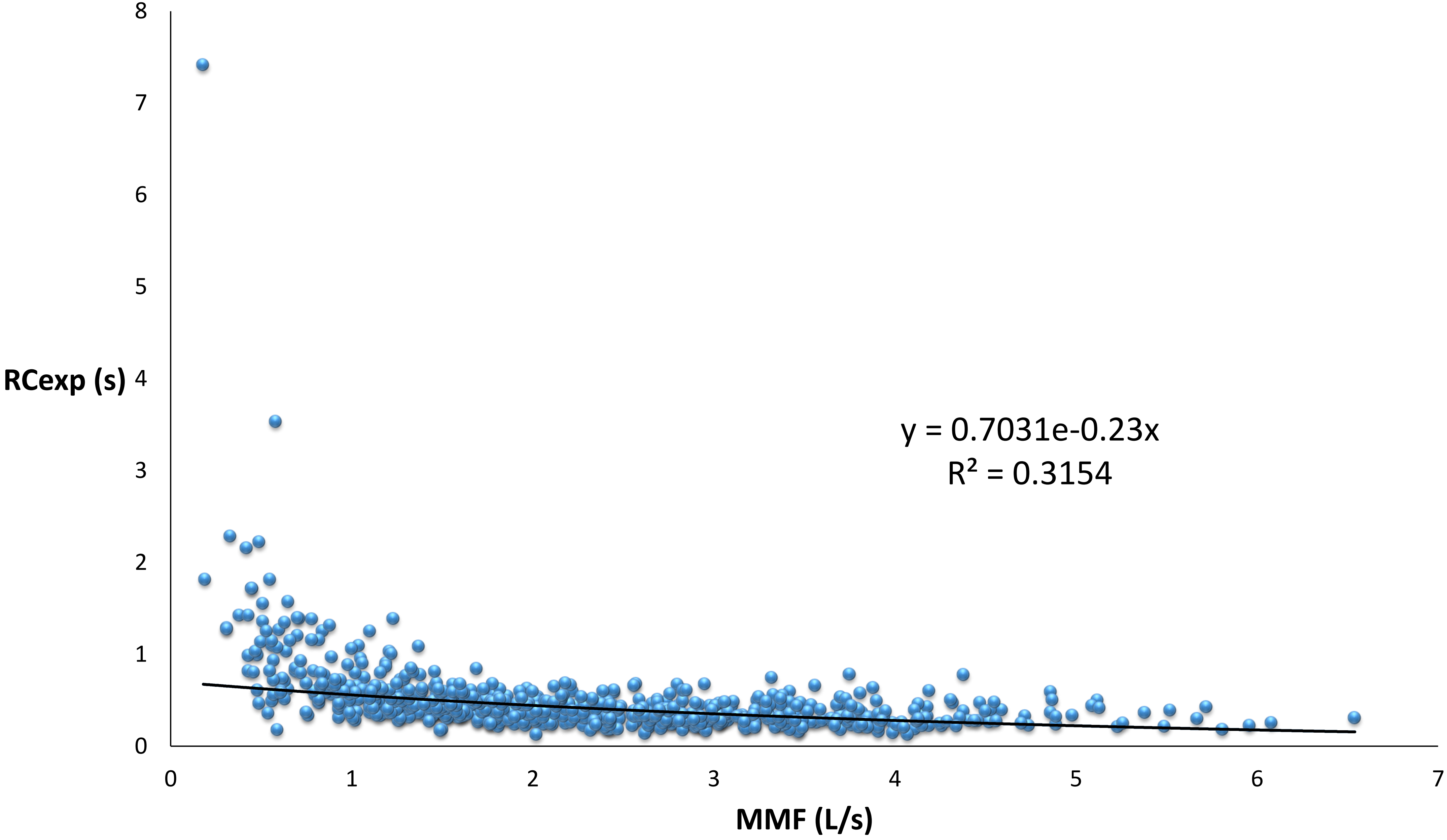

Fig S1. The relationship between RC_{EXP} and MMF.

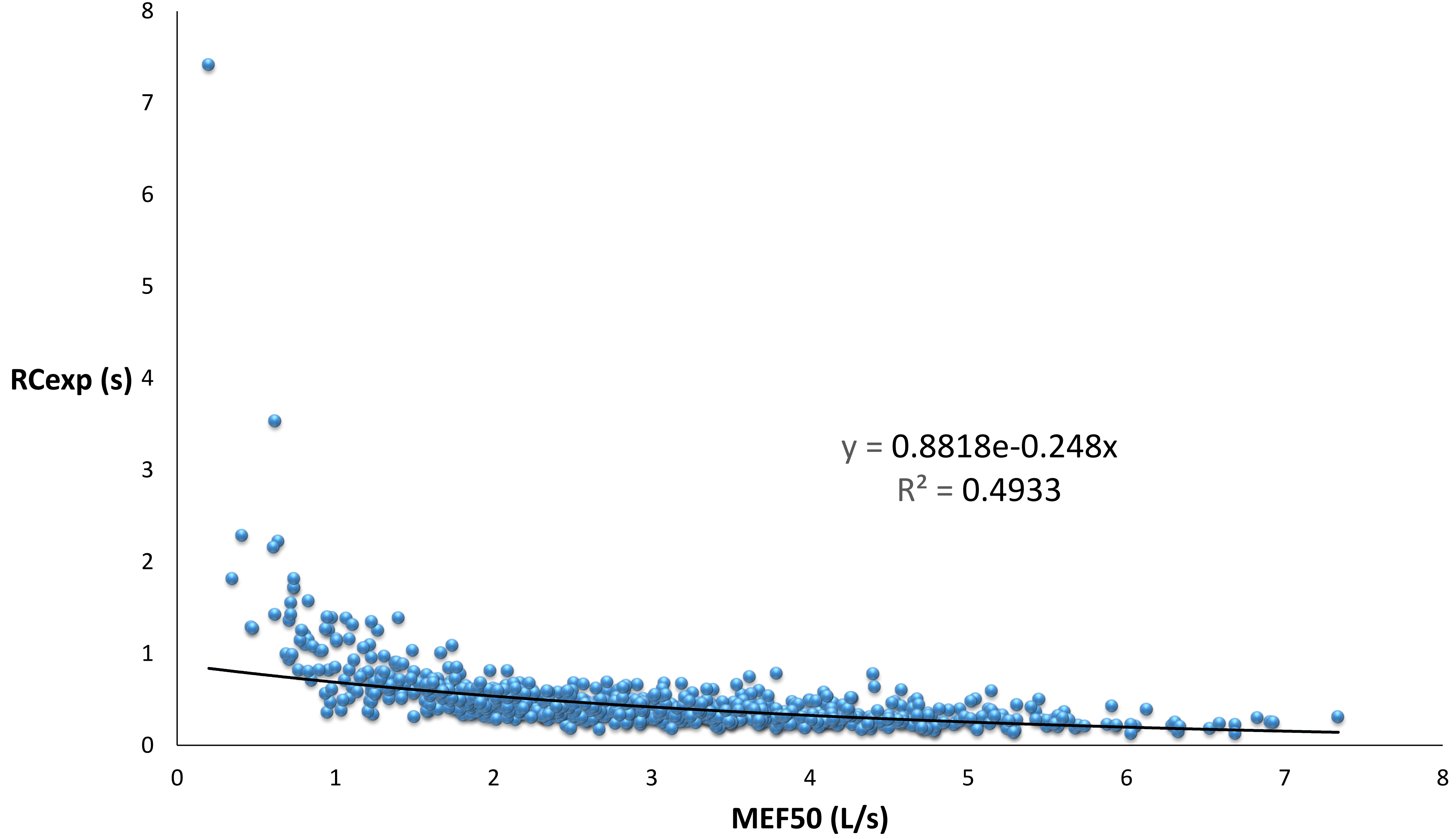

Fig S2. The relationship between RC_{EXP} and MEF_{50}.

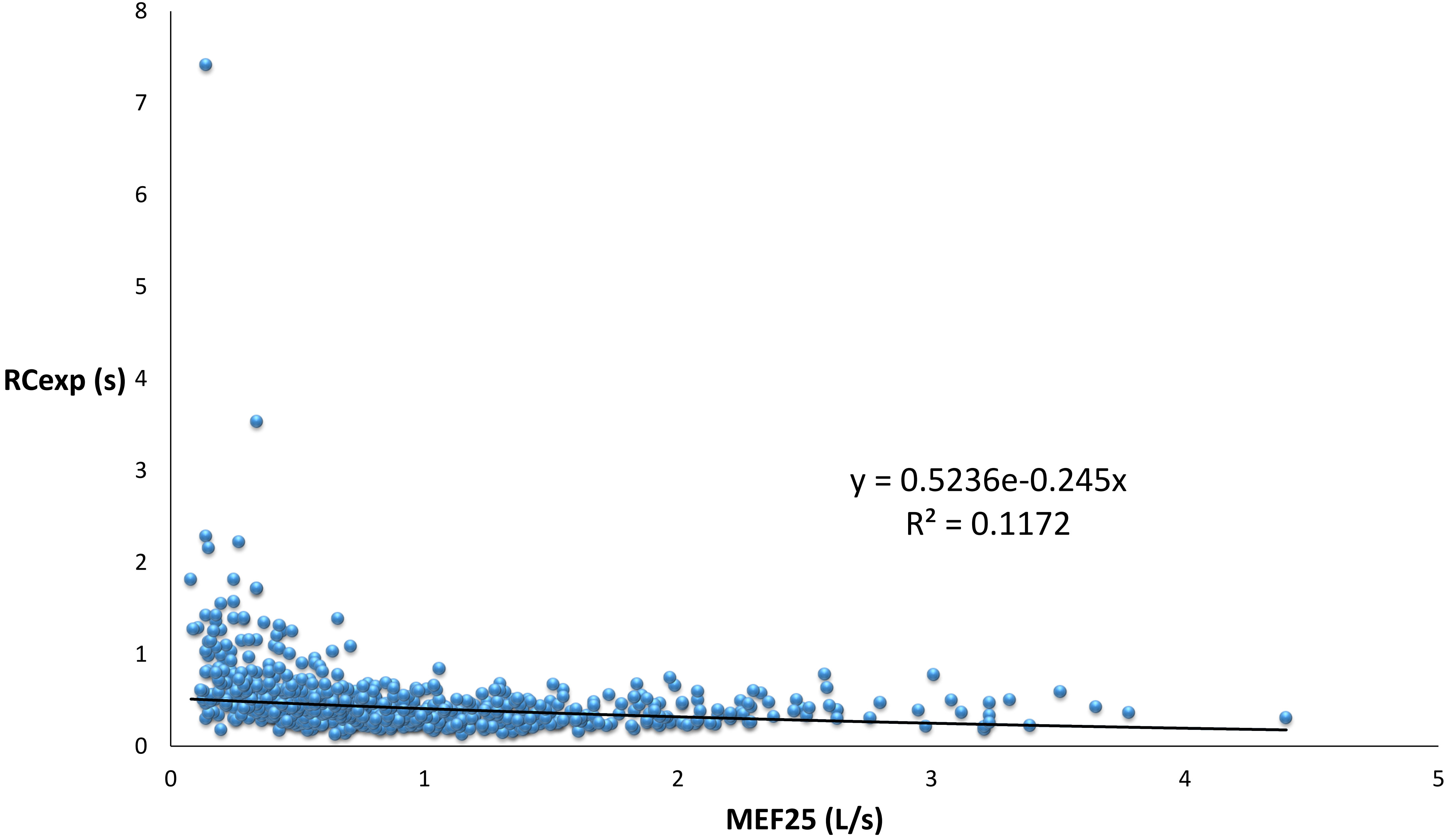

Fig S3. The relationship between RC_{EXP} and MEF_{25}.

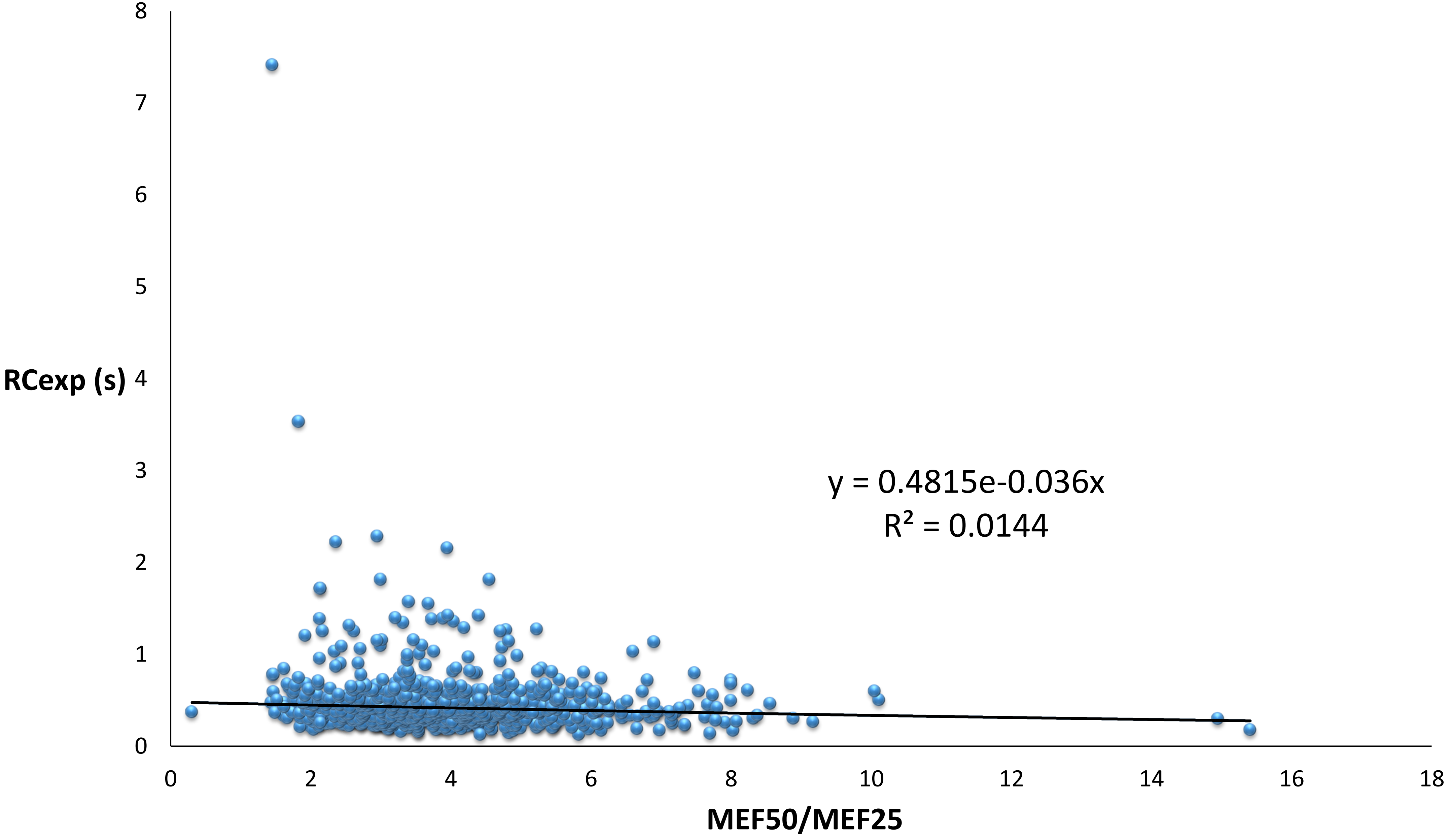

Fig S4. The relationship between RC_{EXP} and MEF_{50}/MEF_{25}.

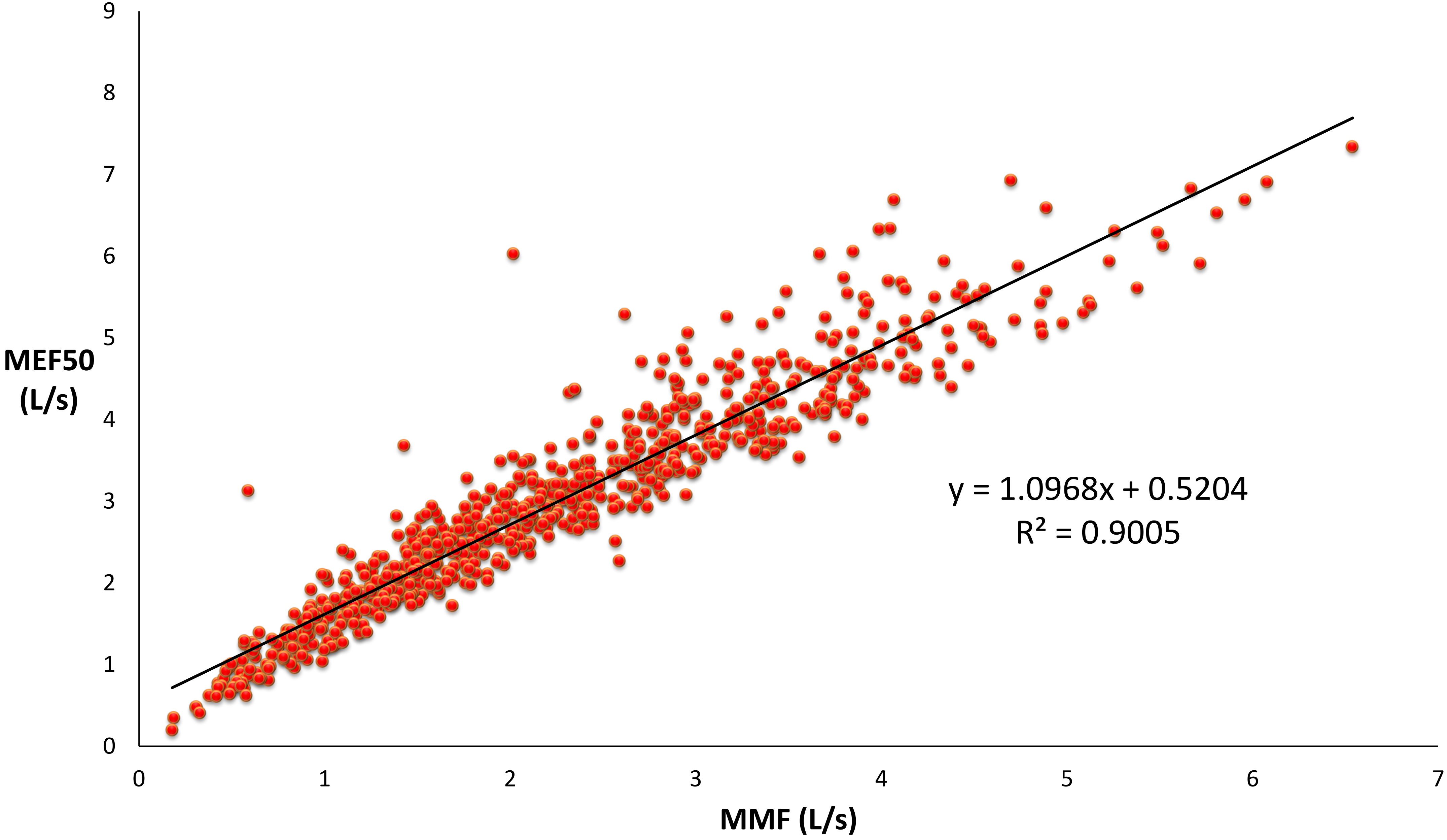

Fig S5. The relationship between MMF and MEF_{50}.

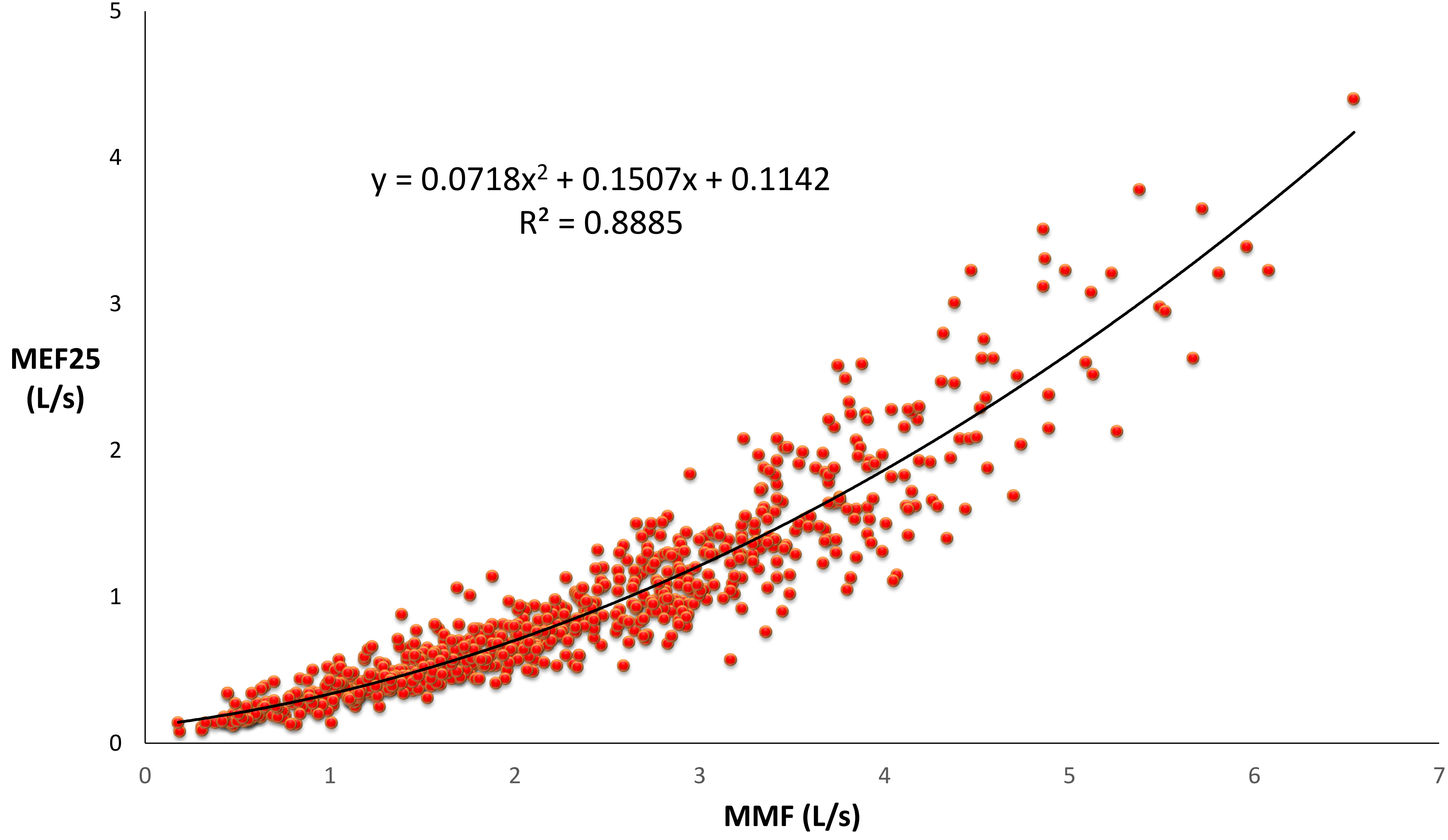

Fig S6. The relationship between MMF and MEF_{25}.
